# Evaluation of the rational prescription of linezolid, the prevalence of thrombocytopenia and major drug interactions in patients with cardiovascular diseases: are there any cautions?

**DOI:** 10.3389/jpps.2024.13343

**Published:** 2024-09-12

**Authors:** Mehrnoush Dianatkhah, Hamed Salami, Rasool Soltani, Alireza Hosseini

**Affiliations:** ^1^ Department of Clinical Pharmacy, Faculty of Pharmacy, Isfahan University of Medical Sciences, Isfahan, Iran; ^2^ Chamran Cardiovascular Medical and Research Hospital, Isfahan, Iran; ^3^ Department of Clinical Pharmacy, Faculty of Pharmacy, Tehran University of Medical Sciences, Tehran, Iran; ^4^ Department of Clinical Pharmacy and Pharmacy Practice, Faculty of Pharmacy, Isfahan University of Medical Sciences, Isfahan, Iran; ^5^ Department of Cardiac Surgery, School of Medicine, Isfahan University of Medical Sciences, Isfahan, Iran

**Keywords:** linezolid, drug interaction, thrombocytopenia, cardiovascular disease, rational prescription

## Abstract

The present study evaluated the rational prescription of linezolid, the prevalence of thrombocytopenia, and major drug interactions in patients with cardiovascular diseases. We conducted a retrospective cross-sectional study on linezolid-treated patients at Shahid Chamran Heart Hospital in Isfahan from March 21, 2021, to March 20, 2022. Our research involved 132 patients who received linezolid. We reported 43.18% of linezolid prescriptions as irrational. Linezolid-induced thrombocytopenia is more common than previous studies, with a prevalence of 47.9%. We found a significant relationship between thrombocytopenia and the concomitant use of aspirin. The duration of treatment was identified as predicting factor for linezolid-induced thrombocytopenia. Moreover, the prevalence of interactions in the X and D categories was determined. Serotonergic and catecholamine medications were associated with 56.1% and 47.7% medication interactions, respectively. Our study found a high prevalence of linezolid-induced thrombocytopenia among patients with cardiovascular diseases. Based on this study, physicians should focus more closely on prescribing linezolid to patients with cardiovascular diseases. In addition to following rational antibiotic use, this susceptible group is also at an elevated risk of side effects (thrombocytopenia) and medication interactions.

## Introduction

Linezolid is one of the most effective antibiotics for treating Gram-positive organisms, including vancomycin-resistant species such as vancomycin-resistant enterococci (VRE), vancomycin-intermediate *Staphylococcus aureus* (VISA), and vancomycin-resistant *S. aureus* (VRSA). In comparison to vancomycin, the use of new-generation antibiotics such as linezolid is increasing due to their advantages, such as high bioavailability for oral administration, fewer allergic reactions, and better tissue penetration. However, linezolid has disadvantages, including thrombocytopenia, a high potential for interaction with catecholamines and serotonergic medications, and a high cost [1–3].

Patients with cardiovascular diseases may be at higher risk for linezolid-induced thrombocytopenia due to the use of anti-platelet and/or anti-coagulant medications [4]. Moreover, these patients may be more susceptible to complications caused by linezolid interactions with adrenergic and/or serotonergic medications, such as hypertension crises, arrhythmias, or heart failure exacerbations [5]. Based on the literature review, no comprehensive investigation has evaluated the prevalence of thrombocytopenia caused by linezolid and its safety in patients with cardiovascular diseases.

This study aims to address the following research questions: (i) What is the prevalence of the rational use of linezolid in people with cardiovascular diseases? (ii) What is the prevalence of significant drug interactions in this patient population? (iii) How prevalent is linezolid-induced thrombocytopenia in patients with cardiovascular diseases?

## Materials and methods

### Participants

We conducted a retrospective cross-sectional study including all the linezolid prescriptions in adult patients (aged >18 years) over a 1-year period at Chamran Heart Hospital in Isfahan from March 21, 2021, to March 20, 2022.

### Data collection

The researchers developed a comprehensive checklist to collect data, which included the following elements: demographics, site and type of infection, microbiological data with related antibiogram, details on linezolid use (start and stop dates, duration of treatment, dose, and route of administration), laboratory data (including serum creatinine, baseline and daily platelet counts), request for consultation with an infectious disease specialist, simultaneous medications, occurrence of thrombocytopenia, and rationality of the prescription according to international guidelines.

### Evaluation of the appropriateness of linezolid prescriptions

Based on international guidelines, including those from IDSA and ESC, two infectious disease specialists independently analyzed the rationality of each prescription. There were four possible reasons for an irrational prescription: no indication for any antibiotic, combinations with a similar spectrum of activity, narrow-spectrum antibiotics possible, and pathogens insensitive to antibiotics. In some cases, linezolid was the only option, while in others, vancomycin was available. Finally, we provided percentage terms for the evaluation of the irrational use of linezolid [6–8].

### Evaluation of linezolid-induced thrombocytopenia

All patients who were treated with linezolid for a minimum duration of 3 days were included in the study. Patients with the following conditions were excluded: patients with a baseline platelet count less than 100 × 10^9^ cells/L prior to therapy, known to have haemato‐oncologic diseases or myelosuppression, who had received bone marrow-suppressing medication within 2 weeks before the study, presence of other diagnoses rather than linezolid-induced thrombocytopenia such as diffuse intravascular disorders (DIC), sepsis, and heparin-induced thrombocytopenia (HIT) with a HIT score above 3. Linezolid-induced thrombocytopenia was defined as a decrease in the patient’s platelet count to less than 100 × 10^9^cells/L or a reduction of more than 25% from their baseline value [9, 10].

To evaluate the relationship between kidney function and the incidence of thrombocytopenia, we divided the patients into three groups according to their creatinine clearance rates (CrCl ≥60 mL/min, 30 mL/min ≤ CrCl <60 mL/min, CrCl <30 mL/min) and compared the prevalence of thrombocytopenia of each group.

### Evaluation of the prevalence of drug interactions

This part of the study included all patients who received linezolid treatment for a minimum of 24 h. Prescriptions with at least two medications, including linezolid were selected for detecting potential drug-drug interactions (DDI) using the UpToDate drug reference database. This database provides information on the mechanism, clinical outcome, and management of possible DDI. The severity of DDI is classified as “(X) avoid combination,” “(D) consider therapy modification,” “(C) monitor therapy,” “(B) no action is needed.” Besides, this reference has classified DDIs based on the clinical severity of the interaction (major, moderate, or minor) and reliability (good and fair). Since potential DDIs in the X and D categories cover most potential DDIs with clinical relevance, we included only these two categories in our analysis [11].

### Statistical analyses

We conducted data analysis using the Statistical Package for Social Sciences version 26.0 software for Windows (IBM SPSS Statistics for Windows, Version 26.0, Armonk, NY: IBM Corp., United States). Kolmogorov-Smirnov tests were employed to assess the normal distribution of continuous variables. We presented the results as mean, standard deviation (SD), median (min-max) for continuous variables, and numbers (percentages) for categorical variables. Furthermore, a chi-square test was used to assess the possible relationship between thrombocytopenia and medications. Logistic regression was used to determine the relationship between thrombocytopenia, age, creatinine clearance (CrCl), baseline platelet counts, and duration of treatment. The possible relationship between basic platelet level and nadir platelet count was assessed by linear regression. The significance level for all tests was set at α < 0.05.

## Results

At Chamran Heart Hospital, 132 patients received linezolid prescriptions during the study period. The demographic characterization of patients including age, gender, route of administration and baseline creatinine clearance are shown in Table 1. The mean age of participants was 65.62 ± 15.82 years (range: 21–98 years), and 71.2% of patients had a CrCl of less than 60 mL/min. The mean linezolid treatment duration was 6.67 ± 5.78 days.

**TABLE 1:** Demographic and clinical characteristics of the included patients.

Variables	N (%) or Mean ± SD
Sex	
Male	91 (68.9)
Female	41 (31.1)
Culture request	
No	45 (34.1)
Yes	87 (65.9)
Rout of administration	
PO	55 (41.7)
IV	77 (58.3)
Duration of treatment	6.67 ± 5.78
CrCl (mL/min)	48.61 ± 26.43
Baseline platelet count (×10^9^ cells/L)	223.6 ± 107.0
Time to thrombocytopenia (days)	6.25 ± 2.93

### Appropriateness of linezolid prescriptions

A total of 132 patients receiving linezolid were included in the evaluation of the rationality of the prescription. Linezolid was most frequently prescribed by infectious specialists (n = 93, 70.5%) and nephrologists (substitution of vancomycin with linezolid in order to avoid vancomycin-induced nephrotoxicity in patients with renal failure) (n = 28, 21.2%), respectively. Linezolid is most commonly used to treat pneumonia. Table 2 shows the prevalence of appropriateness of linezolid prescription in this study. 43.18% of linezolid prescriptions were reported as irrational. There are several reasons for the irrational use of linezolid; however, ignoring culture results and using this antibiotic against insensitive pathogens (such as gram-negative pathogens) was the main one. The antibiotic regimen was not deescalating in the majority of cases, as indicated by the culture results. Moreover, linezolid was the primary treatment option in 13 cases of VRE or VRSA, while vancomycin was another option in 62 cases.

**TABLE 2:** Rational and irrational prescriptions of Linezolid.

Rational/Irrational indications N (%)	Indications	N (%)
Rational use 75 (56.81)	Based on culture (no other options available)	13 (9.84)
	Other options available (mostly vancomycin)	62 (46.96)
Irrational use 57 (43.18)	No indication to any antibiotic	5 (3.87)
	Combination with agents with similar spectrum of activity	3 (3.03)
	Narrower-spectrum antibiotic possible	32 (24.24)
	Insensitive pathogens	17 (12.87.4)

### Prevalence of linezolid-induced thrombocytopenia

This section of the study included 73 patients out of 132 who were receiving linezolid. The following patients were excluded: 28 patients did not have a baseline platelet count, 24 patients were taking linezolid for less than 3 days, 4 patients had a diagnosis of DIC, 2 patients had an HIT score of 4 or higher, and 1 patient was taking carbamazepine concurrently. Out of 73 patients, 35 (47.9%) experienced linezolid-induced thrombocytopenia. Table 3 shows the crude and adjusted models. The results indicated that there was a significant relationship between the occurrence of thrombocytopenia and the duration of treatment (P-value <0.05). Furthermore, upon adjustment, we observed a significant association between the administration of aspirin and the occurrence of thrombocytopenia. According to Table 4, no significant relationship was found between thrombocytopenia and the use of clopidogrel, warfarin, direct oral anticoagulants, enoxaparin, heparin, sex, age, baseline platelet count, and CrCl <60 mL/min.

**TABLE 3:** Univariate Logistic Regression model for risk factors associated with thrombocytopenia.

	Crude Model		Adjusted Model	
Variables	Exp(B) (95%CI)	P	Exp(B) (95%CI)	P
Age	1.01 (0.98–1.04)	0.58	1.02 (0.98–1.06)	0.29
Sex (Female)	0.88 (0.33–2.35)	0.80	0.46 (0.14–1.59)	0.22
Aspirin	2.81 (0.94–8.46)	0.064	4.38 (1.1–17.3)	0.035
Duration of treatment (days)	1.22 (1.07–1.40)	0.002	1.27 (1.10–1.46)	<0.001
Baseline platelet counts (×10^9^ cells/L)	1.00 (0.99–1.00)	0.93	1.00 (0.99, 1.00)	0.93

**TABLE 4:** Risk factors for linezolid‐induced thrombocytopenia.

Medications	Linezolid-induce thrombocytopenia (Yes) N = 35	Linezolid-induce thrombocytopenia (no) N = 38	*P*
Sex (Female)	25 (65.8%)	24 (68.6%)	0.800
Aspirin	29 (82.8%)	24 (63.2%)	0.059
Clopidogrel	11 (31.4%)	15 (39.5%)	0.473
Heparin	11 (31.4%)	19 (50.0%)	0.107
Enoxaparin	4 (11.4%)	6 (15.8%)	0.588
DOAC			0.571
No	31 (88.6%)	35 (92.1%)	
Rivaroxaban	1 (2.9%)	0 (0.0%)	
Apixaban	3 (8.6%)	3 (8.6%)	
Warfarin	10 (28.6%)	9 (23.7%)	0.634
Age (years)	63.4 ± 15.9	61.3 ± 16.4	0.303
Baseline platelet count (×10^9^ cells/L)	225.9 ± 105.0	228.1 ± 120.4	0.93
CrCl (mL/min)	44.6 ± 24.4	47.7 ± 27.5	0.264
Duration (days)	10.7 ± 6.29	6.26 ± 4.01	<0.001

### Prevalence of drug interactions

According to UpToDate drug references, 16.7% of patients had only an X-category interaction, 20.7% had only a D-category interaction, and 34.8% had both interactions concomitantly. The prevalence of drug interactions with serotonergic and catecholamine medications was 56.1% and 47.7%, respectively. A patient who received citalopram and linezolid concomitantly developed serotonin syndrome with fever, agitation, diaphoresis, and tremor that resolved 36 h after both agents were discontinued. The aforementioned patient was receiving 40 mg of citalopram daily, which can be considered high-dose and might justify the occurrence of serotonin syndrome in combination with linezolid. Additionally, one patient who was treated with dobutamine as an inotropic agent developed recurrent ventricular tachycardia, which was subsequently controlled by changing linezolid to vancomycin. The prevalence of interactions between linezolid and serotonergic or catecholamine medications is presented in Table 5.

**TABLE 5:** Interactions between Linezolid and serotonergic and catecholamine medications.

Name	Risk	Severity	Reliability	N (%)
Serotonergic				
Fentanyl	D	Major	Good	33 (25)
Pethidine	D	Major	Good	12 (11)
Methadone	X	Moderate	Fair	13 (9.8)
Acetaminophen codeine	X	Major	Fair	16 (12.1)
Dextromethorphan	X	Major	Fair	5 (3.8)
Morphine	X	Moderate	Fair	31 (23.5)
Sertraline	X	Major	Good	2 (1.2)
Citalopram	X	Major	Good	4 (3)
Escitalopram	X	Major	Good	1 (0.8)
Catecholamine				
Norepinephrine	D	Major	Fair	53 (40.2)
Epinephrine	D	Major	Fair	11 (8.3)
Dopamine	D	Major	Fair	13 (9.8)
Dobutamine	D	Major	Fair	5 (3.8)

## Discussion

In the present study, 43.18% of prescribed cases of linezolid were found to be irrational. Dentan et al. reported this rate as 65% [1]. The majority of irrational use in our study was related to failure to comply with the antibiotic de-escalation strategy after preparation of the antibiogram results. It indicates that the patient’s microbial culture was not adequately considered during the antimicrobial regimen adjustment.

Linezolid was most frequently prescribed by infectious specialists (70.5%), followed by nephrologists (21.2%). Nephrologists substituted all of the prescribed linezolid for vancomycin to prevent vancomycin-induced nephrotoxicity in kidney failure patients requesting nephrology consultations.

Vancomycin alone has a minimal risk of nephrotoxicity (without other risk factors), and recent studies consider this risk to be insignificant. Furthermore, recent review articles indicate the reversible nature of vancomycin-induced acute kidney injury. The toxicity of vancomycin is determined by its minimum concentration; therefore, proper dose adjustment of vancomycin in patients with renal insufficiency, either by adjusting the dose based on serum concentration or by regulating the dose as a whole, can minimize the potential for nephrotoxicity. For this reason, there is no contraindication for the use of vancomycin in patients with renal failure [12, 13].

There is evidence that linezolid-induced thrombocytopenia occurs in 13–30% of patients. In the present study, the prevalence of thrombocytopenia was 47.9%, which was higher compared to other studies. In a retrospective study at Belgian hospital centers conducted by Thirot et al., the prevalence of linezolid-induced thrombocytopenia was reported to be 18.9%. In another study on 254 Chinese patients, the prevalence of linezolid-induced thrombocytopenia was 27.2%. It is possible that the higher prevalence of thrombocytopenia in this study was due to the selection of patients with cardiovascular diseases as the study population, who are more prone to the development of linezolid-induced thrombocytopenia (such as old age, antiplatelet medication use, and chronic kidney disease comorbidities) [2, 13, 14].

Among the antiplatelet and anticoagulant medications, only aspirin showed a statistically significant association with thrombocytopenia. The relationship between linezolid-induced thrombocytopenia and antiplatelet/anticoagulant use is not well studied. Choi et al. found a statistically significant correlation between aspirin and linezolid-induced thrombocytopenia [4]. In the mentioned study, the prevalence of aspirin usage in patients with and without linezolid-induced thrombocytopenia was 82.8% and 63.2%, respectively. The prevalence of linezolid-induced thrombocytopenia was higher among patients with cardiovascular diseases who had received low-dose aspirin. As a result, the prevalence of linezolid-induced thrombocytopenia in our study population (patients with cardiovascular diseases) could be high, which requires closer monitoring of the risk of linezolid-induced thrombocytopenia.

The relation between linezolid-induced thrombocytopenia and low CrCl has been discussed in many studies [15, 16]. Renal failure has been identified as one of the key risk factors for linezolid-induced thrombocytopenia. The morpholine ring of linezolid is mostly oxidized to form two inactive metabolites, and only 30% of the total dose may be excreted in urine as a parent drug. Patients with renal impairment may accumulate two metabolites of linezolid. This correlation (correlation between renal failure and linezolid-induced thrombocytopenia) may result from the accumulation of the culprit metabolite of linezolid (metabolite that suppresses bone marrow cells) in patients with low CrCl [16]. However, in the present study, there was no statistically significant correlation between CrCl and linezolid-induced thrombocytopenia. It appears that our study population’s higher prevalence of renal impairment (71.2% of patients had CrCl <60 mL/min) is responsible for this result. Moreover, the high prevalence of low CrCl in this study might explain the high prevalence of thrombocytopenia. In addition to considering vancomycin-induced nephrotoxicity, it is also important to consider linezolid-induced thrombocytopenia, especially in patients with renal failure [13].

The duration of the treatment had a statistically significant correlation with the occurrence of linezolid-induced thrombocytopenia in our study, which is consistent with findings in other studies. According to Han et al.'s study, the duration of linezolid treatment is associated with linezolid-induced thrombocytopenia [17]. Furthermore, Thirot et al. reported a significant increase in the risk of thrombocytopenia with the use of linezolid for more than 7 days [2].

The current study found that 72.2% of patients had major drug interactions, which can lead to serious complications, especially in patients with cardiovascular disease. Linezolid is a medication with monoamine oxidase inhibitor (MAOI) properties that is able to prevent the metabolism of serotonin, catecholamines, and certain medications in the body. The lack of metabolism of norepinephrine and dopamine in these patients can lead to sympathetic storms, myocardial infarction, ventricular tachycardia, and hypertensive crises. However, its clinical importance has not been investigated. In the heart failure guidelines (AHA/ACC 2022), caution is advised for the use of inotropic and vasopressor agents in combination with MAOIs [5]. In particular, patients with cardiovascular diseases and those at risk of major drug interactions should be carefully monitored when taking linezolid.

Our study reported one case of the serotonin syndrome in a patient who received high dosages of citalopram and linezolid. There has been a report that citalopram and linezolid can cause serotonin syndrome, and citalopram is one of the selective serotonin reuptake inhibitors (SSRIs) with a high prevalence of serotonin syndrome [18]. In addition, a patient who received linezolid and dobutamine developed recurrent ventricular tachycardia. Following the switch from linezolid to vancomycin, the irregular heartbeat improved unexpectedly. A hypothesis can be proposed that linezolid increases serum dobutamine concentration by inhibiting MAO. The clinical effect of linezolid and catecholamine has not been studied, and there is no report about its interaction in clinical studies. Researchers found that when dopamine was co-administered with MAOIs, the amount of dopamine required to elevate systolic blood pressure by 25 mmHg was reduced [19]. According to another study, linezolid administered in conjunction with phenylephrine increased maximum blood pressure by 24 mmHg in healthy volunteers [20]. In combination with linezolid, catecholamines should be initiated at a lower dose and patients should be monitored for an exaggerated hemodynamic response [21].

## Conclusion

This study reported a higher prevalence of thrombocytopenia and potential drug interactions associated with linezolid use in patients with cardiovascular diseases. According to this study, physicians should be more cautious when prescribing linezolid for patients with cardiovascular diseases. This is not only due to the rational use of antibiotics but also due to the higher risk of adverse effects and drug interactions in this vulnerable population.

## Limitations

The retrospective nature of the study limited the ability to comprehensively evaluate certain clinical conditions, such as serotonin syndrome prevalence. There were also limitations including the lack of data recording, such as baseline platelet and serum creatinine levels. In addition, some diagnoses are uncertain in some files. Another significant limitation of the current study is the small sample size, which restricts generalizability. To enhance the robustness and applicability of future studies, it is recommended that a larger sample size and a prospective approach be used.

## Data Availability

The raw data supporting the conclusions of this article will be made available by the authors, without undue reservation.
